# Difference in PaO_2_/FiO_2_ between high-flow nasal cannula and Venturi mask in hypoxemic COVID-19 patients

**DOI:** 10.1186/s44158-022-00051-w

**Published:** 2022-05-24

**Authors:** Ilenia Gatto, Emanuela Biagioni, Irene Coloretti, Serena Viappiani, Stefano Busani, Massimo Girardis

**Affiliations:** Anaesthesiology and Intensive Care Department, University Hospital of Modena, University of Modena and Reggio Emilia, 41125 Modena, Italy

**Keywords:** COVID-19, ICU, HFNC, PaO_2_/FiO_2_

## Abstract

The ratio between arterial blood partial pressure of oxygen and fraction of inspired oxygen (PaO_2_/FiO_2_) was largely used for grading and managing the respiratory failure in non-mechanically ventilated COVID-19. In these patients, the assessment of the true FiO_2_ in the inspired mixture may be difficult with consequent inaccuracies in PaO_2_/FiO_2_ assessment. In 30 severe COVID-19 patients, we observed that PaO_2_/FiO_2_ values measured immediately before and after the transition from high-flow nasal cannula (HFNC) to one commercially available Venturi mask O_2_ therapy were similar (bias mean value 0, standard deviation 23 mmHg). In COVID-19 patients recovering from respiratory failure, PaO_2_/FiO_2_ is not different whether measured with a commercially available Venturi mask or HFNC.

## Introduction

During the SARS-CoV-2 pandemic, the ratio between arterial blood partial pressure of oxygen and fraction of inspired oxygen in the inspired mixture (PaO_2_/FiO_2_, mmHg) was largely used for defining the severity of the respiratory failure and its progression, for deciding the appropriate respiratory support, and, consequently, for using specific pharmacological therapy [1]. Therefore, a careful assessment of PaO_2_/FiO_2_ is fundamental. As it is well known, PaO_2_/FiO_2_ is not the ideal variable for measuring the PO_2_ alveolar-arterial gradient because it does not consider PCO_2_ and its not linear relationship with FiO_2_ regardless of the alveolar-arterial gradient [2]. Moreover, PaO_2_/FiO_2_ was initially proposed in mechanically ventilated patients, where FiO_2_ is carefully measured. In non-mechanically ventilated patients, as patients in conventional O_2_ mask or high-flow nasal cannula (HFNC), the assessment of the true FiO_2_ in the inspired mixture may be problematic, specifically in dyspneic/tachypneic patients with high inspiratory peak flow or with the mouth breathing during HFNC [3, 4]. Therefore, PaO_2_/FiO_2_ values during the transition through the different methods of O_2_ delivery could lead to misinterpretation of the degree of respiratory dysfunction. For the above reasons, we decided to investigate whether the transitions from HFCN and Venturi mask (VM), or vice versa, alter PaO_2_/FiO_2_ values because of potential undetected differences in true FiO_2_.

## Methods

Thirty consecutive patients admitted to our COVID-19 intensive care unit (ICU) because of severe respiratory failure due to SARS-CoV-2 infection and undergoing weaning from respiratory supports were included. As for internal protocol, after weaning from mechanical ventilation and SpO_2_ > 90% with FiO_2_ < 0.7 in HFNC (flow rate 60 L/min), the patients alternated HFNC and VM (FIAB S.p.A., model OS/60K, Florence, Italy) at the same FiO_2_ levels with a progressive de-escalating time scheme. Twenty minutes after the transition from HFNC to VM or vice versa, we collected respiratory rate (RR), mean arterial pressure, heart rate, FiO_2_, PaO_2_, the partial pressure of carbon dioxide (PaCO_2_), and pH in the arterial blood and calculated alveolar-arterial PO_2_ gradient (P(A-a)O_2_) [5]. To evaluate the differences between HFNC and VM, analysis of variance, linear regression analysis, and Bland-Altman method were used [6]. The study was approved by the ethical committee (658/2020/OSS*/AOUMO SIRER ID 417), and informed consent was obtained from participants.

## Results

**Fig. 1:**
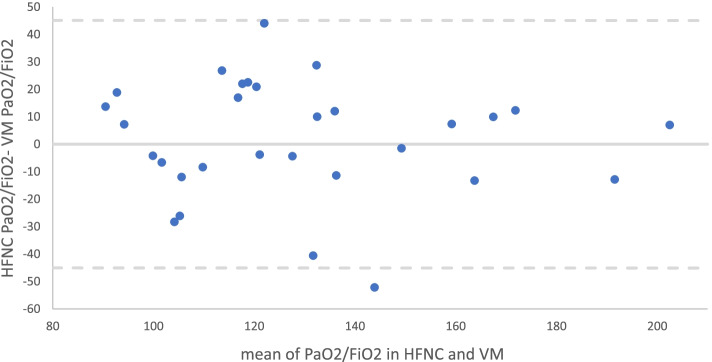
Bland and Altman diagram. The continuous line represents mean bias; the dashed lines represent 1.96 standard deviations. HFNC high-flow nasal cannula, VM Venturi mask

Patients’ characteristics were detailed in Table 1. Twenty-nine patients received HFNC first and only one VM first. The PaO_2_/FiO_2_ measured in HFNC were like those measured in VM (Table 2) with a significant linear relationship (*R*^2^ = 0.51, *p* < 0.01). In the Bland-Altman analysis, the mean value and standard deviation of the bias were 0 and 23 mmHg (Fig. 1). The RR and PaCO_2_ values resulted to be different (*p* = 0.013 and *p* = 0.001) with RR higher and PaCO_2_ lower in VM compared to HFNC (Table 2). In patients previously treated with HFNC, PaCO_2_ was lower during VM in 25 patients (86.2%), and the difference was up to 5 mmHg in only 4 patients (13.8%).

**Table 1:** Clinical characteristics of the patients

Age, years (median, IQR)	63 (53–72)
Sex, male (*n*, %)	17 (56,7)
First test HFNC (*n*, %)	29 (96,7)
ICU LOS prior test (days, median, IQR)	5 (3–8)
IMV prior test (*n*, %)	16 (53,3)
NIV prior test (*n*, %)	23 (76,7)
IMV length prior test (days, median, IQR)	2 (0–6)
NIV length prior test (days, median, IQR)	1 (0–2)
RASS score during HFNC (median, IQR)	0
RASS score during VM (median, IQR)	0

*HFNC* high-flow nasal cannula, *ICU LOS* length of stay in intensive care unit, *IMV* invasive mechanical ventilation, *NIV* non-invasive mechanical ventilation, *RASS* Richmond Agitation-Sedation Scale, *VM* Venturi mask

**Table 2:** Blood gases values and vital signs in HFNC and VM

	HFNC	VM	*p*-value
PaO_2_/FiO_2_ (mmHg)	127 (100–147)	118 (107–152)	0.604
PaO_2_ (mmHg)	59.0 (55.0–64.8)	60.0 (50.2–70.0)	0.906
P (A-a) O_2_ (mmHg)	236 (196–266)	242 (203–264)	0.122
FiO_2_	0.50 (0.45–0.50)	0.50 (0.40–0.50)	0.180
SaO_2_ (%)	91.10 (88.40–93.40)	92.10 (87.00–94.20)	0.688
PaCO_2_ (mmHg)	39.20 (34.70–42.10)	36.60 (32.00–40.90)	0.001
pH	7.49 (7.46–7.51)	7.50 (7.47–7.52)	0.323
RR (breaths/min)	21 (17–25)	23 (20–28)	0.013
MAP (mmHg)	90 (87–93)	90 (87–95)	0.311
HR (beats/min)	75 (60–85)	80 (64–90)	0.189

Data are presented as median (IQR). *RR* respiratory rate, *MAP* median arterial pressure, *HR* heart rate, *HFNC* high-flow nasal cannula, *VM* Venturi mask, *PaO*_*2*_/*FiO*_*2*_ arterial blood partial pressure of oxygen and fraction of inspired oxygen, *PaO*_*2*_ arterial blood partial pressure of oxygen, *P (A-a) O*_*2*_ alveolar-arterial PO_2_ gradient, *FiO*_*2*_ fraction of inspired oxygen, *SaO*_*2*_ saturation of oxygen, *PaCO*_*2*_ partial pressure of carbon dioxide

## Discussion

Our data indicate that after 20 min from HFNC to VM transition and vice versa, PaO_2_/FiO_2_ remain similar with only a small and not significant difference. Previous studies suggested that HFNC may improve oxygenation and decrease work of breathing compared to conventional O2 therapy [7–9]. In contrast with previous reports, our results may suggest that there may be an underestimation of FiO_2_ with Venturi device yielding overestimation of PaO_2_/FiO_2_ in contrast with the accurate delivery of FiO_2_ with HFNC. Anyway, in this specific population under these circumstances, both these two phenomena have clinically acceptable limits of agreement. Moreover, we observed that RR differed between the two methods despite the very short period of exposure (20 min). The main part of the sample transitioned to VM after having received HFNC, so it cannot exclude a possible carry-over effect for PaCO_2_ in this transition. Moreover, a possible carry-over effect cannot be excluded also for PaO_2_/FiO_2_ ratio. Rather than reducing work of breathing, the association of lower RR with a consensual increase of PaCO_2_ supports the hypothesis that HFNC, compared to VM, could improve oxygenation with less requirement of alveolar ventilation for maintaining PaO_2_ level. However, the low number of patients and the lack of assessment of the true FiO_2_ limit any further speculation on this point.

In conclusion, our study demonstrated that although in HFNC and VM the FiO_2_ is only estimated and may vary with the patient's respiratory pattern, PaO_2_/FiO_2_ measured with a VM may be considered a reliable parameter for respiratory dysfunction evaluation in severely hypoxemic patients during the transitions from different O_2_ delivery methods.

## Data Availability

The datasets used and/or analyzed during this study are available from the corresponding author on reasonable request.

## Abbreviations

HFNC: High-flow nasal cannula
VM: Venturi mask
ICU: Intensive care unit
RR: Respiratory rate
P(A-a)O_2_: Alveolar-arterial PO_2_ gradient
MAP: Median arterial pressure
HR: Heart rate
PaO_2_/FiO_2_: Arterial blood partial pressure of oxygen and fraction of inspired oxygen
